# Correction: Evolving a 24-hr oscillator in budding yeast

**DOI:** 10.7554/eLife.07532

**Published:** 2015-03-27

**Authors:** Gregg A Wildenberg, Andrew W Murray

Wildenberg GA, Murray AW. 2014. Evolving a 24-hr oscillator in budding yeast. *eLife* **3**:e04875. doi: 10.7554/eLife.04875. Published 10 November 2014

In the published article, Figure 4A listed 8 mutated genes incorrectly. In the text, we referred to the causative alleles of the mutated genes by their allele names (e.g., *sir4-100*), but in Figure 4A we mistakenly used the nature of the mutations (e.g., *sir4-L1012P*) as their allele names. This figure has been corrected to give both the allele names and the nature of each mutation.

Figures 4B, 4C and 4D are correct and unchanged from the original published figure, and we apologize for the original mistake.

The corrected Figure 4 is shown below:

**Figure 1:**
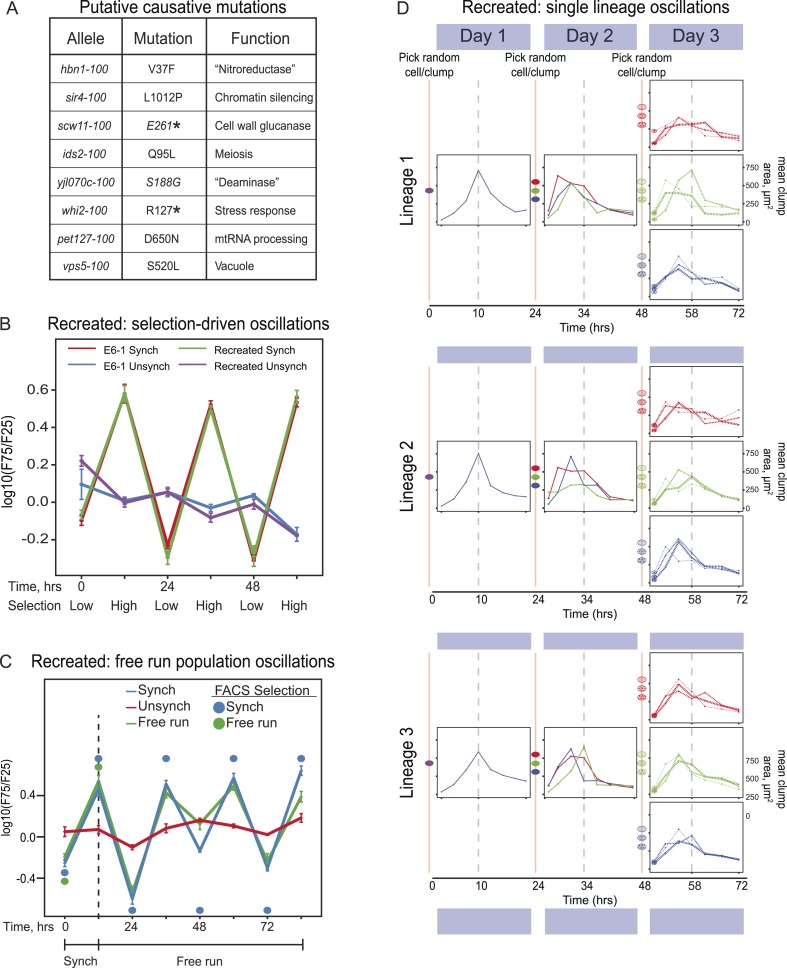

The originally published Figure 4 is also shown for reference:

**Figure 2:**
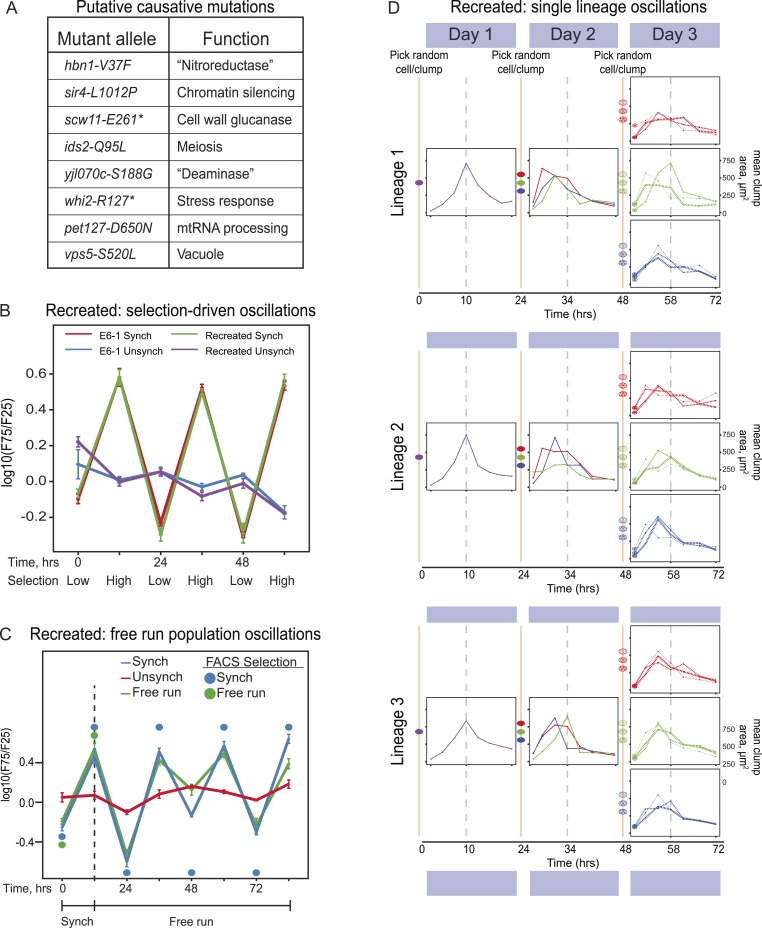

The article has been corrected accordingly.

