# Supplementary material for: Evolving a 24-hr oscillator in budding yeast
Source: eLife. 2014 Nov 10;3:e04875. doi: 10.7554/eLife.04875 (PMC4270073; doi:10.7554/eLife.04875)
Supplement: Supplementary file 1. — Table of the strains used and their relevant genotypes. DOI: http://dx.doi.org/10.7554/eLife.04875.014 [file elife04875s003.docx]

Wildenberg, Murray Supplementary File 1

| **­­Strain name** | **Genetic change** | **Additional Information** |
| --- | --- | --- |
| yGW 461 | MAT**a** *BUD4* *HIS3*∆::*P_ACT1_*-ymCherry-*tADH1*-*HIS3MX6* *P_FLO1_*::*P_FLO1_*-ymCitrine-*tADH1* *pol3-L523D leu2-3,112 ura3∆0 can1-100* | Ancestor |
| yGW E6 | Ancestor genotype: evolved 360 generations | Evolved population: 6% selection |
| yGW E15 | Ancestor genotype: evolved 360 generations | Evolved population: 15% selection |
| yGW 5A7 | Ancestor genotype: evolved 360 generations | E6-1 evolved clone from 6% evolved population |
| yGW 501 | *P_ACT1_*-ymCerulan-*tADH1* | E6-1 + CFP |
| yGW 556 | MATalpha lys2∆::Hygro^r^ | Mating type switched Ancestor used for crossing to E6-1 and bulk segregant analysis |
| yGW 451 | Ancestor with wild type POL3 | Lab strain used to introduce clock alleles: direct descendant of ancestor prior to introduction of *pol3-L523D* |
| yGW 992 | MAT**a** BUD4 *HIS3*∆::P_ACT1_-ymCherry-tADH1- *HIS3*∆::MX6 *P_FLO1_*::*P_FLO1_*-ymCitrine-tADH1 *leu2-3,112 ura3∆0 can1-100 hbn1 ids2 pet127 scw11 sir4 vps5 whi2-100 yjl070c* | Complete recreated clock strain with all 8 mutations |
| yGW 861 | *hml∆::Ca.URA3* | E6-1 with *hml*∆ |
| yGW 1A1 | Ancestor genotype: evolved 360 generations | E6-2 clone |
| yGW 903 | *hml∆::Ca.URA3* | E6-2 hml∆ |
| yGW 1G1 | Ancestor genotype: evolved 360 generations | E6-3 clone |
| yGW 905 | *hml∆::Ca.URA3* | E6-3 *hml*∆ |
| yGW 5A1 | Ancestor genotype: evolved 360 generations | E6-4 clone |
| yGW 900 | *hml∆::Ca.URA3* | E6-4 *hml*∆ |
| yGW 5C5 | Ancestor genotype: evolved 360 generations | E6-5 clone |
| yGW 898 | *hml∆::Ca.URA3* | E6-5 *hml*∆ |
| yGW 1B2 | Ancestor genotype: evolved 360 generations | E15-1 clone |
| yGW 896 | *hml∆::Ca.URA3* | E15-1 *hml*∆ |
| yGW 1C3 | Ancestor genotype: evolved 360 generations | E15-2 clone |
| yGW 894 | *hml∆::Ca.URA3* | E15-2 *hml*∆ |
| yGW 1D10 | Ancestor genotype: evolved 360 generations | E15-3 clone |
| yGW 907 | *hml∆:: Ca.URA3* | E15-3 *hml*∆ |
| yGW 1E7 | Ancestor genotype: evolved 360 generations | E15-4 clone |
| yGW 893 | *hml∆::Ca.URA3* | E15-4 *hml*∆ |
| yGW 936 | *hbn1∆:: KAN^r^* | E6-1 *hbn1*∆ |
| yGW 932 | *ids2∆:: KAN^r^* | E6-1 *ids2*∆ |
| yGW 924 | *pet127∆:: KAN^r^* | E6-1 *pet127*∆ |
| yGW 926 | *scw11∆:: KAN^r^* | E6-1 *scw11*∆ |
| yGW 928 | *sir4∆:: KAN^r^* | E6-1 *sir4*∆ |
| yGW 934 | *vps5∆:: KAN^r^* | E6-1 *vps5*∆ |
| yGW 923 | *whi2-100∆:: KAN^r^* | E6-1 *whi2*∆ |
| yGW930 | *yjl070c∆:: KAN^r^* | E6-1 *yjl070c*∆ |
| yGW E6/1A9 | Ancestor genotype: evolved 360 generations | E6-6 |
| yGW E6/5A5 | Ancestor genotype: evolved 360 generations | E6-7 |
| yGW E6/1B8 | Ancestor genotype: evolved 360 generations | E6-8 |
| yGW E6/1B12 | Ancestor genotype: evolved 360 generations | E6-9 |
| yGW E6/1C3 | Ancestor genotype: evolved 360 generations | E6-10 |
| yGW E6/1D10 | Ancestor genotype: evolved 360 generations | E6-11 |
| yGW E6/1H6 | Ancestor genotype: evolved 360 generations | E6-12 |
| yGW E6/5A11 | Ancestor genotype: evolved 360 generations | E6-13 |
| yGW E6/5B4 | Ancestor genotype: evolved 360 generations | E6-14 |
| yGW E6/5B8 | Ancestor genotype: evolved 360 generations | E6-15 |
| yGW E6/5B12 | Ancestor genotype: evolved 360 generations | E6-16 |
| yGW E15/1A7 | Ancestor genotype: evolved 360 generations | E15-5 |
| yGW E15/1E1 | Ancestor genotype: evolved 360 generations | E15-6 |
| yGW E15/1E3 | Ancestor genotype: evolved 360 generations | E15-7 |
| yGW E15/1F6 | Ancestor genotype: evolved 360 generations | E15-8 |
| yGW E15/1G9 | Ancestor genotype: evolved 360 generations | E15-9 |
| yGW E15/2A1 | Ancestor genotype: evolved 360 generations | E15-10 |
| yGW E15/2B6 | Ancestor genotype: evolved 360 generations | E15-11 |
| yGW E15/2C7 | Ancestor genotype: evolved 360 generations | E15-12 |
| yGW E15/2D8 | Ancestor genotype: evolved 360 generations | E15-13 |
| yGW E15/2E3 | Ancestor genotype: evolved 360 generations | E15-14 |
| yGW E15/2F6 | Ancestor genotype: evolved 360 generations | E15-15 |
